# Rowell Syndrome with Good Response to Methotrexate

**DOI:** 10.31138/mjr.33.1.92

**Published:** 2022-03-31

**Authors:** Madhuri Challa, Ritasman Baisya, Phani Kumar Devarasetti

**Affiliations:** Clinical Immunology & Rheumatology Nizam’s Institute of Medical Sciences (NIMS), Hyderabad, India

## PRESENTATION

**Figure 1.**
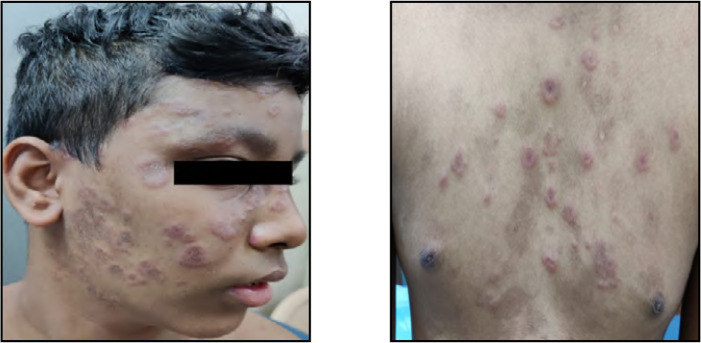
Extensive erythema multiform-like lesion, with target appearance with lupus erythematous rash over face and anterior aspect of chest.

**Figure 2.**
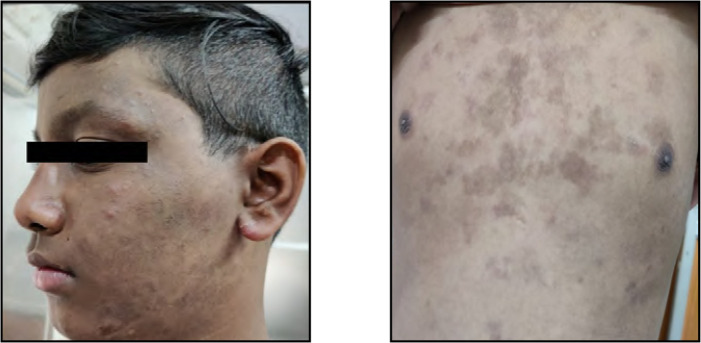
Healing of lesions after treatment.

A ten-year-old boy presented to Rheumatology OPD with a short history of polyarthritis, constitutional symptoms, and a generalised rash all over the body. Physical examination revealed well-defined, erythematous papules and plaques, mostly annular with target appearance associated with scales on the face, chest, abdomen. Few of them merged into large, confluent, arcuate lesions. His investigation showed Antinuclear antibody 4 (+) speckled, Anti-Smith antibody, Anti-ribonucleoprotein (RNP) antibody, IgM- Rheumatoid Factor, anti-Ro antibody (ELISA) positive. Anti-dsDNA antibody was negative. His clinical diagnosis fulfilled the 2000 revised diagnostic criteria for Rowell syndrome by Zeitouni et al.^1^ After treatment with low dose steroids, hydroxychloroquine, and methotrexate, his rashes were improved within a few weeks. (**Figures 1 and 2**).

## DISCUSSION

In 1963, Rowell et al.^2^ described this syndrome characterised by lupus erythematosus (LE) and erythema multi-forme (EM)-like lesions. Subsequently, in 2000, major and minor diagnostic criteria were proposed for the diagnosis of this syndrome by Zeitouni et al.^1^ Major criteria include coexistence of LE and EM-like lesions, and positive ANA with a speckled pattern. Minor criteria include chilblains, positive anti-La (SS-B) or anti-Ro (SS-A) antibodies, and reactive rheumatoid factor. All the major criteria and at least one of the minor criteria are required to confirm the diagnosis. The present case fulfilled all major and two minor criteria satisfying the diagnosis of Rowell syndrome.
